# The effects of seeding density and osteoclastic supplement concentration on osteoclastic differentiation and resorption

**DOI:** 10.1016/j.bonr.2022.101651

**Published:** 2022-12-21

**Authors:** Stefan J.A. Remmers, Freek C. van der Heijden, Keita Ito, Sandra Hofmann

**Affiliations:** Orthopaedic Biomechanics, Department of Biomedical Engineering and Institute for Complex Molecular Systems, Eindhoven University of Technology, Eindhoven, the Netherlands

**Keywords:** Iβ3, integrin β3, M-CSF, macrophage colony-stimulating factor, PBMC, peripheral blood mononuclear cell, pNPP, (4-nitrophenyl) dihydrogen phosphate, RANKL, receptor activator of nuclear factor kappa-B ligand, TRAP, tartrate resistant acid phosphatase, Osteoclast, Monocyte, PBMC, Resorption, TRAP, Priming

## Abstract

The bone resorbing osteoclasts are a complex type of cell essential for *in vivo* bone remodeling. There is no consensus on medium composition and seeding density for *in vitro* osteoclastogenesis, despite the importance thereof on osteoclastic differentiation and activity. The aim of this study was to investigate the relative effect of monocyte or peripheral blood mononuclear cell (PBMC) seeding density, osteoclastic supplement concentration and priming on the *in vitro* generation of functional osteoclasts, and to explore and evaluate the usefulness of commonly used markers for osteoclast cultures. Morphology and osteoclast formation were analyzed with fluorescence imaging for tartrate resistant acid phosphatase (TRAP) and integrin β3 (Iβ3). TRAP release was analyzed from supernatant samples, and resorption was analyzed from culture on Corning® Osteo Assay plates. In this study, we have shown that common non-standardized culturing conditions of monocyte or PBMCs had a significant effect on the *in vitro* generation of functional osteoclasts. We showed how increased osteoclastic supplement concentrations supported osteoclastic differentiation and resorption but not TRAP release, while priming resulted in increased TRAP release as well. Increased monocyte seeding densities resulted in more and large TRAP positive bi-nuclear cells, but not directly in more multinucleated osteoclasts, resorption or TRAP release. Increasing PBMC seeding densities resulted in more and larger osteoclasts and more resorption, although resorption was disproportionally low compared to the monocyte seeding density experiment. Exploration of commonly used markers for osteoclast cultures demonstrated that Iβ3 staining was an excellent and specific osteoclast marker in addition to TRAP staining, while supernatant TRAP measurements could not accurately predict osteoclastic resorptive activity. With improved understanding of the effect of seeding density and osteoclastic supplement concentration on osteoclasts, experiments yielding higher numbers of functional osteoclasts can ultimately improve our knowledge of osteoclasts, osteoclastogenesis, bone remodeling and bone diseases.

## Highlights

• Increasing supplement concentrations increased osteoclastogenesis and resorption.
• TRAP release should not be used as a quantitative marker for resorption.
• Integrin β3 is an excellent osteoclast marker that is more specific than TRAP.
• There is an optimal monocyte seeding density for osteoclastic resorption.
• The use of monocytes leads to disproportionately more resorption than PBMCs.

## 1 Introduction

Osteoclasts are bone resorbing cells, that together with bone forming osteoblasts and regulating osteocytes are responsible for bone homeostasis. Monocytes are the main osteoclast precursors (Udagawa et al., 1990), and circulate in the peripheral blood (Henriksen et al., 2012; Kleinhans et al., 2015) as a portion of the peripheral blood mononuclear cells (PBMCs). Monocytes are recruited by osteoblasts and osteocytes (Graves et al., 1999; Kim et al., 2006) towards the site of resorption where they differentiate through cell-fusion to form osteoclasts through biochemical signaling of receptor activator of nuclear factor kappa-Β ligand (RANKL) and macrophage colony stimulating factor (M-CSF) (Takahashi et al., 1999; Teitelbaum, 2000; Yoshida et al., 1990).

Much is still unknown about osteoclasts. Their limited availability, complex manner of attraction and differentiation, high between-donor variation (Flanagan and Massey, 2003; Husch et al., 2021) and short lifespan (Manolagas and Parfitt, 2010; Parfitt, 1994) make it challenging to study them. The use of immortalized cell lines such as murine RAW 264.7 (Collin-Osdoby and Osdoby, 2012) or human THP-1 (Hayden et al., 2014) partially mitigates the availability, between-donor variation and lifespan concerns. At the same time however, their unnatural immortality introduces a lifetime deviation from the *in vivo* situation, and they neglect the variation found in patients or healthy donors which ultimately limits translatability of the obtained results. The use of animal cells shares similar concerns for translation towards human health and disease (Burkhardt and Zlotnik, 2013; Contopoulos-Ioannidis et al., 2003). To study human bone remodeling, ideally human primary cells should be used (Owen and Reilly, 2018).

*O*steoclasts are defined as resorbing, multinucleated (≥3 nuclei), tartrate resistant acid phosphatase (TRAP) expressing cells with a clearly defined actin ring (Buckley et al., 2005). TRAP, an enzyme secreted in the ruffled border, has been the dominant osteoclast marker for decades and has been linked to lacunar ATP hydrolysis, reactive oxygen species generation, and *in vivo* bone turnover (Hayman, 2008; Hayman et al., 1996; Kaunitz and Yamaguchi, 2008). However, TRAP expression is not exclusive to osteoclasts, as it is expressed in other cells (Hayman et al., 2001) including macrophages (Lord et al., 1990) who share a common monocyte precursor. More recently integrin β3 (Iβ3), an NF-κB-associated cell-surface receptor (Antonov et al., 2011) with a role in actin ring formation (Lee et al., 2015) has been used as an osteoclast marker as well (Barbeck et al., 2017; Nakamura et al., 2007). While TRAP is expressed both in mononuclear cells and in multinucleated osteoclasts, integrin expression changes from monocyte/macrophage marker β2 to β3 upon differentiation towards osteoclasts (Husch et al., 2021).

Contrary to what the name suggests, osteoclast cultures do not generally develop into pure populations of osteoclasts. Instead, these cultures facilitate the differentiation of precursors into relatively low numbers of osteoclasts, while still containing many precursors throughout the culture. While many studies using osteoclast cultures have been done in the last decades, there is no consensus on the various parameters that should be used for *in vitro* research such as seeding densities, medium composition and osteoclastic supplement concentrations (Remmers et al., 2021). However, it is generally agreed upon that so called ‘priming’ of monocytes with M-CSF for a certain duration before the addition of RANKL has a beneficial effect on osteoclastogenesis (De Vries et al., 2015; Ross, 2006; Xu and Teitelbaum, 2013) in part because of its stimulatory effect on the expression of various genes including RANKL-related tumor necrosis factor (TNF) (Hayes and Zoon, 1993; Lacey et al., 1998; Yasuda et al., 1998). A recent systematic map of osteoblast-osteoclast co-cultures showed that commonly used seeding densities ranged from 5 to 250 × 10^3^ cells/cm^2^, and supplement concentrations of RANKL and M-CSF ranged from 10 to 100 ng/mL (Remmers et al., 2021). The large variation is surprising, considering that culture medium content (Mather, 1998; Shahdadfar et al., 2005) and cell seeding density (Kozbial et al., 2019) greatly affect osteoclastogenesis. Combined with the short lifespan of osteoclasts and the complex manner of obtaining them in meaningful numbers, these factors pose significant challenges to the design and execution of cell-culture experiments. A better understanding of the effect of these parameters on osteoclastogenesis, osteoclastic activity and resorption would result in higher yields of functional osteoclasts, ultimately improving our knowledge of osteoclasts, osteoclastogenesis, bone remodeling and bone diseases.

The aim of this study was to investigate the relative effect of osteoclastic supplement concentration (RANKL and M-CSF) and seeding density on osteoclastogenesis and functionality. Additionally, we evaluated the usefulness of commonly used tests and markers for osteoclast cultures.

## 2 Materials and methods

### 2.1 Materials

Two human buffy coats were obtained from Sanquin (Eindhoven, Netherlands) after review and approval of the study by the Sanquin ethics review board. The buffy coats were collected by Sanquin under their institutional guidelines and with written informed consent per Declaration of Helsinki. Fetal Bovine Serum (FBS, batch F7524-500ML/lot BCBV7611) was from Sigma Aldrich/Merck (Zwijndrecht, The Netherlands). RPMI-1640 medium was from Thermo Fisher Scientific (Breda, The Netherlands). Antibiotic/antimycotic (anti-anti) was from Life Technologies (Bleiswijk, The Netherlands). Lymphoprep™ was from Axis-Shield (Oslo, Norway). MACS® Pan Monocyte Isolation Kit was from Miltenyi Biotec (Leiden, the Netherlands). Recombinant human M-CSF and recombinant human RANKL were from PeproTech (London, United Kingdom). Antibody Iβ3 (Orb248939, Mouse monoclonal) was from Biorbyt (Cambridge, United Kingdom). Antibody TRAP (Sc-30833, Goat polyclonal) was from Santa-Cruz Biotechnology, Inc. (Heidelberg, Germany). Antibody Alexa 488 (715-545-150, Donkey-anti-Mouse IgG H + L) was from Jackson ImmunoResearch (Cambridgeshire, United Kingdom). Antibody Alexa 488 (A11055, Donkey-anti-Goat IgG H + L) was from Molecular Probes (Eugene, OR, USA). Antibody Alexa 350 (A10035, Donkey-anti-Mouse IgG H + L) was from Invitrogen (Waltham, MA, USA). Thin bleach was from the local Jumbo grocery store (Stiphout, Netherlands). All other substances were of analytical or pharmaceutical grade and obtained from Sigma Aldrich/Merck (Zwijndrecht, The Netherlands).

### 2.2 Methods

#### 2.2.1 Monocyte isolation

**Supplementary Fig. S1:**
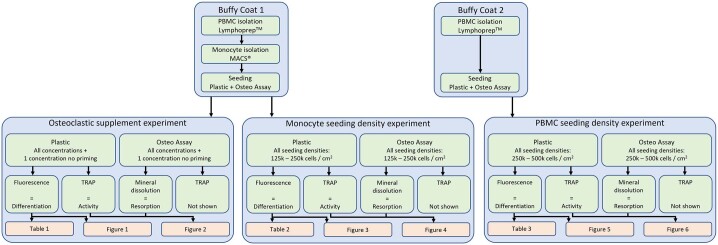
Schematic overview of methods and analyses applied to each buffy coat

Two human peripheral blood buffy coats from two separate healthy donors were obtained from the local blood donation center under informed consent. The buffy coats (±50 mL each) were processed independently in a similar manner as described previously (Bonito et al., 2019; Remmers et al., 2020). In short, they were diluted to 180 mL in sodium citrate dissolved in phosphate buffered saline (PBS) (0.6 % *w*/*v*) adjusted to pH 7.2 at 4 °C (citrate buffer). PBMCs were isolated by layering onto Lymphoprep™ isoosmotic medium (30 mL diluted buffy coat onto 16 mL Lymphoprep™) and centrifuging at 800 × *g* for 30 min (lowest brake and acceleration). The PBMC layer was extracted using sterile Pasteur pipettes and PBMCs were washed 5 × with citrate buffer (350 × *g* for 10 min) to remove any remaining Lymphoprep™. PBMCs were used as is for one buffy coat, or further processed to isolate monocytes using the negative selection MACS® Pan Monocyte Isolation Kit as specified by the manufacturers' instructions for the other buffy coat. The Pan Monocyte isolation kit was chosen specifically to isolate a larger mixed population of classical, non-classical and intermediate subtype monocytes in favor of a single subtype, because all subtypes have been shown capable of osteoclastic differentiation (Yao et al., 2021). Here, non-monocytes were labeled with magnetic microbeads and retained in a filter column (size LS) in a magnetic field, while unlabeled cells passed through and were collected to be used as monocytes in this study. A schematic overview of the methods and analyses applied to each of the two buffy coats is shown in supplementary Fig. S1.

#### 2.2.2 Variation in RANKL and M-CSF supplement concentration

250 × 10^3^ monocytes per cm^2^ (n = 4 per group) were seeded in priming medium (De Vries et al., 2015) (RPMI-1640, 10 % FBS, 1 % Anti-Anti, 50 ng/mL M-CSF) on 24-well Corning® Osteo Assay plates and regular 24-well tissue culture plates in monolayer. Priming medium was replaced after 48 h with base medium (RPMI-1640, 10 % FBS, 1 % Anti-Anti) supplemented with 0, 12.5, 25, 50 or 100 ng/mL of both M-CSF and RANKL. Additionally, one group was seeded without priming medium and cultured directly in medium containing 50 ng/mL of both supplements. Medium was replaced 3 × per week for 2 weeks. All wells received the same medium volume per medium change (1 mL per well). In the remainder of this study, unless otherwise stated, RANKL and M-CSF are meant when (osteoclastic) supplements are indicated.

#### 2.2.3 Variation in monocyte/PBMC seeding density

Monocytes were seeded at densities of 125, 150, 175, 200, 225 and 250 × 10^3^ cells/cm^2^, and PBMCs were seeded at densities of 250, 300, 350, 400,450 and 500 × 10^3^ cells/cm^2^ (*n* = 4 per group) in priming medium on 24-well Corning® Osteo Assay plates and regular 24-well tissue culture plates in monolayer. PBMCs were seeded at a higher density to partially compensate for the fact that only approximately 20 % of PBMCs are monocytes (Kleiveland, 2015). Priming medium was replaced after 48 h with osteoclast medium (RPMI-1640, 10 % FBS, 1 % Anti-Anti, 50 ng/mL M-CSF and RANKL). Medium was replaced 3 × per week for 2 weeks. All wells received the same medium volume per medium change (1 mL per well).

#### 2.2.4 TRAP release quantification

Samples of cell culture supernatant were taken prior to each medium change and stored at −80 °C. 20 μL sample or p-nitrophenol standard in assay buffer (0.1 M sodium acetate, 0.1 % (v/v) Triton X-100 in PBS adjusted to pH 5.5) were incubated for 90 min at 37 °C with 100 μL para-nitrophenylphosphate (pNPP) buffer (1 mg/mL pNPP + 30 μL/mL tartrate solution in assay buffer). Stop solution (100 μL 0.3 M NaOH in ultra-pure water) was used to stop the reaction after 90 min. Absorbance was measured at 405 nm with a Synergy HTX Multi-Mode microplate reader (BioTek, Winooski, United States).

#### 2.2.5 Immunofluorescent labelling and cell counting

The cells cultured in plastic well-plates were fixed in 10 % neutral buffered formalin for 10 min, permeabilized with 0.5 % Triton X-100 in PBS for 10 min, blocked with 10 % horse serum in PBS for 30 min, washed with wash buffer (50 nM Tris pH 7.4, 150 mM NaCl, 5 mM EDTA, 0.05 % NP-40, 0.25 % gelatin). Cells were labeled with one of three marker combinations: TRAP + actin + DAPI with Alexa 488 donkey-anti-goat secondary antibody, Iβ3 + actin + DAPI with Alexa 488 donkey-anti-mouse secondary antibody, or TRAP + Iβ3 + actin with Alexa 488 donkey-anti-goat and Alexa 350 donkey-anti-mouse antibodies. The cells were incubated with primary antibodies for osteoclast markers TRAP and/or Iβ3 overnight at 4 °C (1:100 in wash buffer +10 % horse serum). The next day, the cells were washed with wash buffer and incubated for 1 h with the respective secondary antibodies (1:300), TRITC-conjugated-Phalloidin (1:200) for actin and DAPI (1:1000) for nuclei in wash buffer. The cells were washed with PBS and imaged with a thin layer of PBS in the wells. Fluorescence images were taken with a Axiovert 200 M microscope (Carl Zeiss Microscopy, Oberkochen, Germany). From each well, an image of the center of the well was used for image analysis with ImageJ (Schneider et al., 2012), resulting in 3 images per group. All Iβ3-positive cells with ≥3 nuclei were counted within the field of view. Of these cells, the number of nuclei and longest diameter were recorded, together with the total number of nuclei in the images.

#### 2.2.6 Matrix dissolution on Osteo assay plates

Corning® Osteo Assay plates were analyzed in a similar manner as described elsewhere (Ren et al., 2019; Sinnesael et al., 2015). Cells on the Corning® Osteo Assay plates were removed using thin bleach (5 %) for 5 min. The wells were washed with ultra-pure water and dried at 50 °C. Bright field images were taken with a Axio Observer Z1 microscope (Carl Zeiss Microscopy, Oberkochen, Germany). Multiple images were stitched together to cover the complete surface area of the wells. An area of 3 by 5 stitched images completely within the well was cropped and used for image analysis. Images were binarized manually or in small batches using a combination of Matlab R2022a, Ilasik version 1.0 (Berg et al., 2019), and ImageJ (Schneider et al., 2012) with Fiji (Schindelin et al., 2012) to create as accurate as possible binarizations. From these, the percentage of matrix dissolution (hereafter referred to as resorption) was calculated. Staining was not necessary for this method. An area of 3 by 5 stitched images was selected to improve Ilastik machine learning and binarization results, because light and well-shape artifacts around the edges of the wells obstructed the machine learning algorithm. A pixel classification algorithm was used in Ilastik, where areas of resorption and unresorbed areas were manually labeled to teach Ilastik how to recognize these in the current and subsequent images. The result was visually judged per image, and the algorithm was corrected and reapplied as necessary.

#### 2.2.7 Statistical analysis

Quantitative data is represented as mean ± standard deviation (SD) and was analyzed using GraphPad Prism version 8. Where applicable, data used for statistical analysis was tested for normality using the Shapiro-Wilk normality test and was normally distributed. Resorption data was analyzed with a Student's *t*-test or a One-Way Analysis of Variances (ANOVA). TRAP release data was compared using a Repeated Measures ANOVA. Fluorescence image analysis results were compared using a Repeated Measures ANOVA and ANOVA for trend analysis. Bonferroni correction was used to account for multiple *post-hoc* comparisons. Geisser-Greenhouse correction was used to account for unequal variances. Differences were considered statistically significant at a level of *p* < 0.05. Where multiple comparisons were listed as a single difference, only the least significant (highest) *p* value was listed. Throughout this study, notable significant effects were numbered in the results section, figures and tables using unique sequential numbering signed with a ‘*’ or ‘^#^’ for figures and tables respectively.

## 3 Results

### 3.1 Osteoclastic supplement concentration affected osteoclast generation, TRAP release and resorption in monocytes

**Fig. 1:**
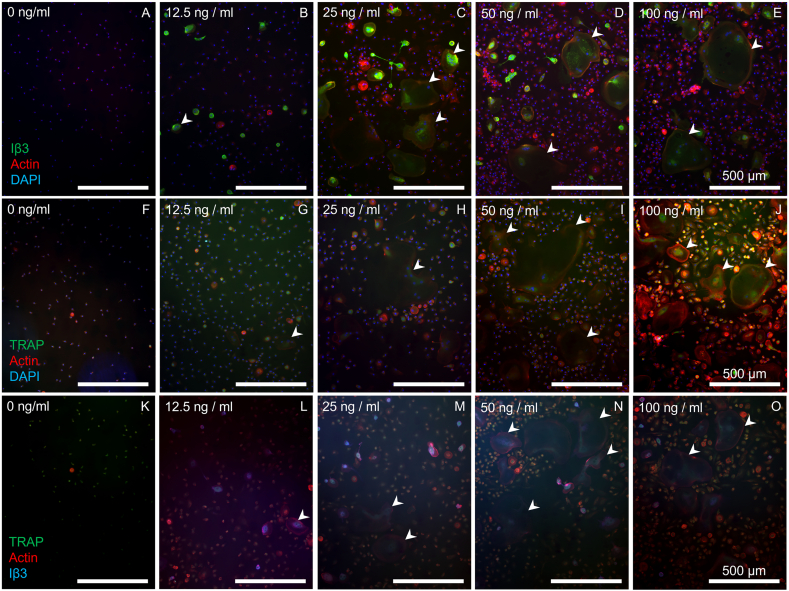
The size and number of osteoclasts increases as osteoclastic supplement concentrations increased in monocyte cultures. Monocytes were seeded at different densities, cultured, and stained in one of three ways to compare TRAP and Iβ3 stains. (A – E) Iβ3, actin and DAPI staining showed that RANKL and M-CSF are required for Iβ3 expression. At 12.5 ng/mL, Iβ3-positive cells were present, but remain small. At 25 ng/mL and higher, Iβ3-positive cells appeared larger and more numerous. (F – J) TRAP, actin and DAPI staining showed minor TRAP expression but no evidence of multinucleated cells at 0 ng/mL. Multinucleated TRAP-positive cells were seen at 12.5 ng/mL, but only at 25 ng/mL they became larger with many more nuclei. Concentrations higher than 25 ng/mL seemed to result in even more TRAP expression. (K – O) TRAP, actin and Iβ3 staining showed combined TRAP-Iβ3 expression predominantly in larger and multinucleated cells. Examples of multinucleated TRAP or Iβ3 positive multinucleated cells were indicated with white arrowheads.

Monocytes were cultured in culture media with one of 6 concentrations of RANKL and M-CSF after a common priming phase of 2 days. Fluorescence imaging showed that at 0 ng/mL, minor TRAP expression was detected in monocytes, but Iβ3 expression or multinucleated cells were not (Fig. 1a, f, k). TRAP and Iβ3 positive multinucleated cells were detected at 12.5 ng/mL where all Iβ3-positive cells were small. Increased osteoclastic supplement concentrations led to more and larger multinucleated cells (Fig. 1b–e, g–j, l–o), but from 25 ng/mL and higher, this did not lead to further significant increases in number, nuclei count or size of osteoclasts (Table 1).

**Table 1:** Osteoclast quantification with different supplement concentrations. Osteoclasts are here defined as Iβ3-positive cells with ≥3 nuclei and were analyzed in 3 images per group. Results are shown ± SD. Osteoclast numbers, nuclei per osteoclast and average diameter all show a significant increasing trend (*p* < 0.001) with increasing supplement concentration. After correction for multiple comparisons however, the number of osteoclasts, nuclei per osteoclast and average diameter do not significantly change at concentrations equal or higher than 25 ng/mL. The number of osteoclasts in the 100 ng/mL group is only significantly different from the 0 and 12.5 ng/mL group (^#^1: *p* ≤ 0.037), while the average diameter of the 12.5 ng/mL group is significantly different from all other groups with identified osteoclasts (^#^2: *p* ≤ 0.0229).

Supplement concentration	0 ng/mL	12.5 ng/mL	25 ng/mL	50 ng/mL	100 ng/mL
Osteoclasts per image	0^#^1	1.0 (±0.0)^#^1	4.7 (±2.9)	5.3 (±2.1)	6.7 (±2.1)^#^1
Nuclei per osteoclast	N.A.	3.0 (±0)	4.9 (±1.6)	7.6 (±4.7)	5.2 (±3.1)
Average diameter (μm)	N.A.	75 (±10) ^#^2	268 (±96)	354 (±160)	268 (±174)
Total nuclei per image	119 (± 57)	278 (±200)	322 (±73)	540 (±240)	581 (±45)

**Fig. 2:**
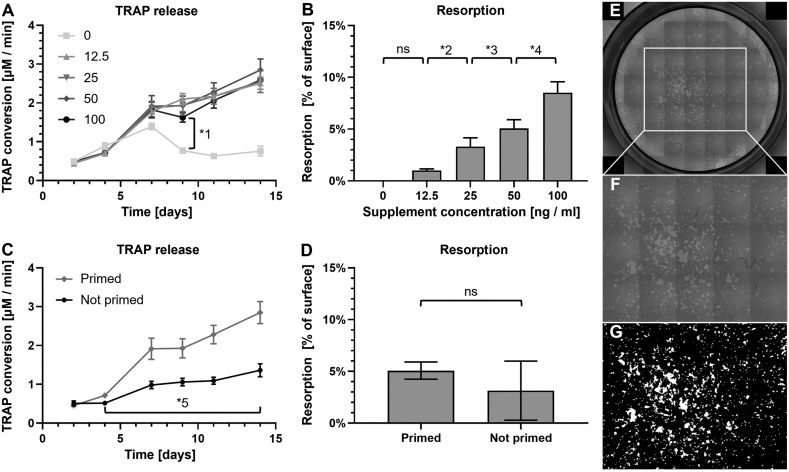
Osteoclastic supplement concentration affected resorption but not TRAP release in monocyte culture. (A) TRAP release increased near-equally in all groups except the 0 ng/mL group, which from day 9 onward deviated from all groups (*1: *p* ≤ 0.024). (B) Higher osteoclastic supplement concentrations resulted in more resorption after 14 days, showing a significant linear trend over all data (*p* < 0.001). The difference between 0 and 12.5 ng/mL was not significant (ns) (*p* = 0.64). All other differences were statistically significant (*2: *p* = 0.004, *3: *p* = 0.032, *4 *p* < 0.001). (C) TRAP release of the primed group was significantly higher than that of the not primed group from day 4 onward (*5: *p* ≤ 0.024). (D) Resorption analysis shows a substantial but not statistically significant difference of 1.93 percentage points (*p* = 0.241), with a much smaller SD in the primed group compared to the group without priming. (E) Resorption in an Osteo Assay well, imaged with light microscopy and stitched together. The 3 images by 5 images area used for analysis is shown in the white rectangle. Resorption is visible in white/light grey on an unresorbed dark grey background. (F) An enlargement of the area that was used for image analysis. (G) A binarized image of the selected area of interest, which allows easy quantification of the resorbed areas in white and the unresorbed areas in black.

TRAP release analysis showed that TRAP release by monocytes increased sharply in all groups until day 7 (Fig. 2a). As expected, the 0 ng/mL group stopped releasing TRAP after this initial release that was likely caused by the priming phase. In all other groups, TRAP release continued and increased until the end of culture. Remarkably, TRAP release per well was similar for all concentrations except the 0 ng/mL group. Resorption data showed only resorption after culture with any non-zero concentration of added osteoclastic supplements (Fig. 2b). No resorption took place without these supplements, even though all cultures were primed for 2 d. Increasing osteoclastic supplement concentrations resulted in more resorption.

Priming of monocytes resulted in more TRAP release (Fig. 2c) and resorption (Fig. 2d) compared to the control group without priming. The unprimed group was cultured directly in 50 ng/mL of both osteoclastic supplements and showed considerably less resorption than the primed 50 ng/mL group but similar resorption as the 25 ng/mL group. While the observed difference was substantial, it was not statistically significant. An example of fully imaged Osteo Assay well, the area selected for analysis and the binarized image thereof are shown in Fig. 2e–g respectively.

### 3.2 Monocyte seeding density affected osteoclastic differentiation, but not TRAP release or resorption

**Fig. 3:**
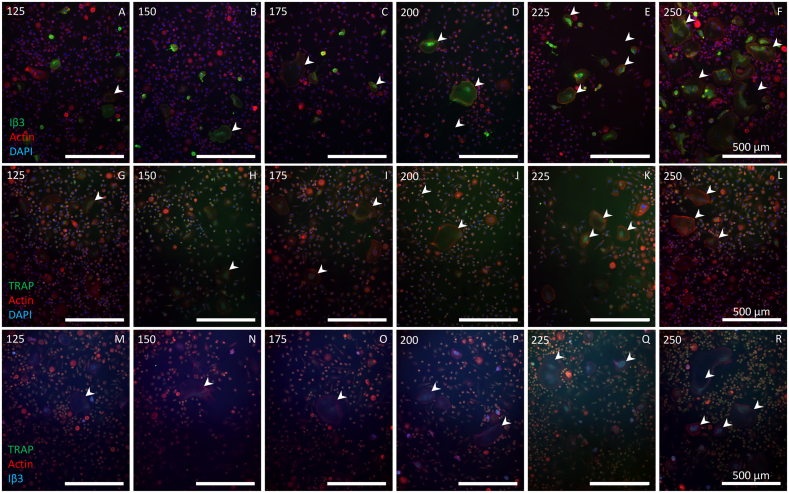
Higher monocyte seeding density led to slightly more and larger osteoclasts. Monocytes were seeded at different seeding densities and stained in one of three ways. (A – F) Iβ3, actin and DAPI staining showed that all seeding densities resulted in Iβ3-positive cells. The size and number thereof increased as seeding density increased. (G – L) TRAP, actin and DAPI staining showed that TRAP positive cells were abundant in all seeding densities. While the size of TRAP positive cells increased with seeding density, the number of TRAP positive cells only increased marginally. (M – R) TRAP, actin and Iβ3 staining showed that not all TRAP positive cells were also positive for Iβ3. Iβ3 was almost exclusively seen in the larger or multinucleated cells whereas TRAP was also seen in many small mononuclear cells as well. Numbers in the top left corners indicate the monocyte seeding density × 10^3^ cells/cm^2^. Examples of multinucleated TRAP and/or Iβ3 positive cells are indicated using white arrowheads.

Monocytes were seeded at 6 densities (125, 150, 175, 200, 225 and 250 × 10^3^ cells/cm^2^) to investigate how different seeding densities affect multinuclear cell generation, resorption, and TRAP release. TRAP- and Iβ3-positive cells were seen at all seeding densities. The number and size of large Iβ3-positive cells increased slightly with seeding density, although many contained only two nuclei (Fig. 3). The size and nuclei count of Iβ3-positive cells with ≥3 nuclei were similar at all seeding densities, only increasing at the highest density (Table 2).

**Table 2:** Osteoclast quantification with different monocyte seeding densities. Osteoclasts are here defined as Iβ3-positive cells with ≥3 nuclei and were analyzed in 3 images per group. Results are shown ± SD. The number of osteoclasts, nuclei per osteoclast or average diameter of osteoclasts did not significantly increase with increasing seeding densities. After correction for multiple comparisons, only the diameter of the 125 and 175 × 1000 cells/cm^2^ groups were significantly different (^#^3: *p* = 0.013).

Monocyte seeding density (× 1000 cells/cm^2^)	125	150	175	200	225	250
Osteoclasts per image	2.3 (±3.2)	0.0	1.0 (±1.0)	1.3 (±1.2)	1.3 (±0.6)	7.3 (±7.8)
Nuclei per osteoclast	3 (±0.0)	N.A.	4 (±1.7)	3.5 (±0.6)	3.5 (±1.0)	7.5 (±3.4)
Average diameter (μm)	143 (±90)^#^3	N.A.	277 (±136)^#^3	237 (±75)	197 (±19)	234 (±81)
Total nuclei per image	536 (±120)	512 (±115)	223 (±112)	280 (±87)	366 (±240)	727 (±36)

**Fig. 4:**
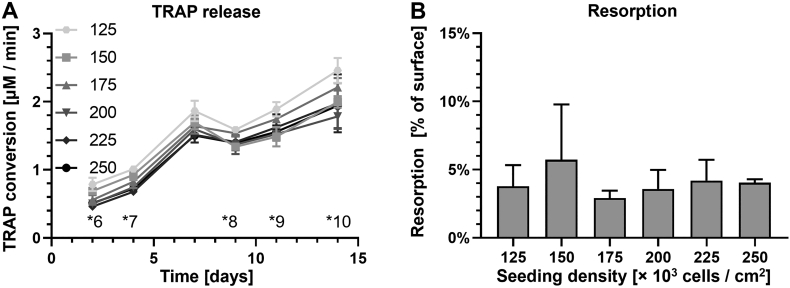
Monocyte seeding density hardly affected total TRAP release and did not affect resorption. (A) TRAP release over time increased in all seeding densities and seemed to be higher in the lower seeding densities. There were some significant differences that were inconsistent over time between the groups with one notable exception: the 125 × 10^3^ cells/cm^2^ group was significantly different from at least one of the 200, 225 and 250 × 10^3^ cells/cm^2^ groups on all indicated significances (*6 - *10, *p* < 0.05). (B) Resorbed surface area at different seeding densities. No significant differences in resorption were observed.

Unexpectedly, TRAP release results (Fig. 4a) suggested that there is very little difference in TRAP release between monocyte seeding densities. Remarkably, there were some significant differences between the 125 and 150 × 10^3^ cells/cm^2^ groups *vs.* 200, 225 and 250 × 10^3^ cells/cm^2^ groups, suggesting that lower seeding densities resulted in higher TRAP release.

Resorption analysis matched these findings and showed that similar amounts of resorption occurred for all monocyte seeding densities (Fig. 4b), while increased seeding densities were expected to result in more resorption. The highest amount of resorption was measured in the 150 × 10^3^ cells/cm^2^ group, one of the lowest seeding densities, although none of the differences between groups were statistically significant.

### 3.3 PBMC seeding density affected multinuclear cell generation and resorption, but hardly affected TRAP release

**Fig. 5:**
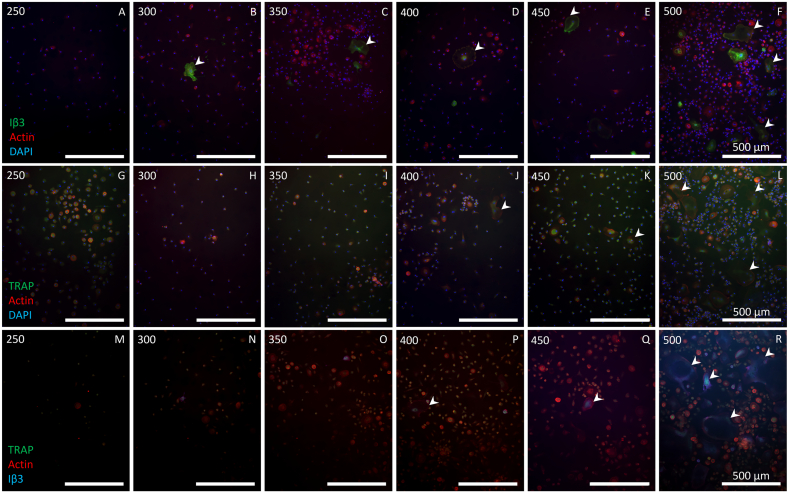
Higher PBMC seeding density led to more osteoclasts. PBMCs were seeded at different densities and stained in one of three ways. (A – F) Iβ3, actin and DAPI staining showed that all but the lowest seeding density resulted in Iβ3-positive cells. At seeding densities from 300 to 450 × 10^3^ cells/cm^2^, only few Iβ3-positive cells were seen. Only at the highest seeding density there were many Iβ3-positive cells. (G – L) TRAP, actin and DAPI staining showed that nearly all cells expressed TRAP, but only at a seeding density of 400 × 10^3^ cells/cm^2^ or higher, larger multinucleated TRAP-positive cells were seen, with their numbers and size increasing at higher seeding densities. (M – R) TRAP, actin and Iβ3 staining showed that not all TRAP positive cells were also positive for Iβ3. Iβ3 was sporadically seen until 450 × 10^3^ cells/cm^2^, and only at 500 × 10^3^ cells/cm^2^ Iβ3 was present in many cells. Numbers in the top left corners indicate the PBMC seeding density × 10^3^ cells/cm^2^. Examples of multinucleated TRAP or Iβ3 positive cells are indicated by white arrowheads.

PBMCs were seeded at 6 densities (250, 300, 350, 400, 450 and 500 × 10^3^ cells/cm^2^) to investigate how different seeding densities affected multinuclear cell generation, TRAP release and resorption. Fluorescence imaging revealed that TRAP expression was present at all seeding densities (Fig. 5g–r). While there were no clearly visible multinucleated TRAP positive cells until a PBMC seeding density of 400 × 10^3^ cells/cm^2^ (Fig. 5j), Iβ3-positive cells were already seen starting at 300× 10^3^ cells/cm^2^ (Fig. 5b) although these did not contain ≥3 nuclei (Table 3). Only at the highest seeding density, a higher number of large multinucleated cells was observed (Fig. 5l + r), although most of these contained only 2 nuclei and the number of cells with ≥3 nuclei remained low.

**Table 3:** Osteoclast quantification with different PBMC seeding densities. Osteoclasts are here defined as Iβ3-positive cells with ≥3 nuclei and were analyzed in 3 images per group. Results are shown ±SD. * = based on n = 2 due to a corrupt file. Statistical analysis was not possible for the PBMC experiment, because not enough wells contained identified osteoclasts.

PBMC seeding density (× 1000 cells/cm^2^)	250	300	350	400*	450	500
Osteoclasts per image	0	0	0	0.3 (±0.6)	0	1.3 (±2.3)
Nuclei per osteoclast	N.A.	N.A.	N.A.	3.0 (±0.0)	N.A.	5.3 (±1.9)
Average diameter (μm)	N.A.	N.A.	N.A.	204 (±0)	N.A.	159 (±82)
Total nuclei per image	60 (±15)	174 (±84)	260 (±129)	169 (±74)	110 (±48)	407 (±345)

**Fig. 6:**
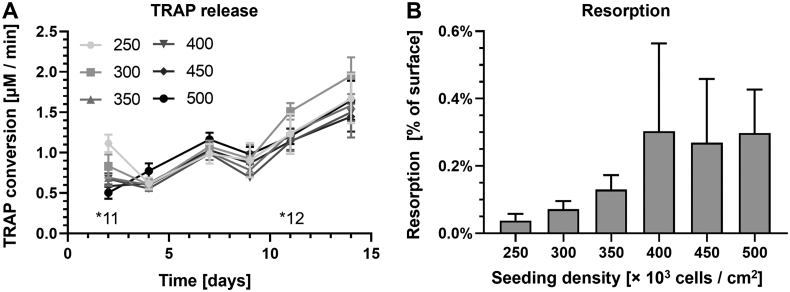
Increased PBMC seeding densities hardly affected TRAP release but seemed to increase total resorption. (A) TRAP release over time increased in all seeding densities and seemed to be higher in the lower seeding densities during week 2 of culture. Significant differences were inconsistent over the duration of the culture. *11: 250 *vs.* 350, 400, 450 & 500 (*p* ≤ 0.021). *12: 300 *vs.* 400 (*p* = 0.031). (B) Increased PBMC seeding densities seemed to lead to increased resorption. The linear trend that is visible is not significant (*p* = 0.079). While some differences were statistically significant, these significances disappeared after correcting for multiple comparisons.

TRAP release showed no large differences between the seeding densities (Fig. 6a). On two occasions, the 250 and 300 × 10^3^ cells/cm^2^ (*11 and *12) groups released significantly more TRAP than higher seeding densities, but these effects were not consistently present. Although remarkably, lower seeding densities seemed to release slightly more TRAP than higher seeding densities. This was in line with the observations made in the monocyte seeding density experiment.

Resorption correlated with seeding density, and gradually increased as seeding density increased (Fig. 6b). Despite the high resorption of the 400 × 10^3^ cells/cm^2^ group with large SD, resorption followed a linear trend that was almost significant (*p* = 0.079). While there were clear differences between the resorption of the seeding densities and a trend is evident, none of these were significant after *post hoc* Bonferroni correction for multiple comparisons.

## 4 Discussion

The limited *in vitro* availability, complex manner of differentiation, high between-donor variation (Flanagan and Massey, 2003; Husch et al., 2021) and short lifespan (Manolagas and Parfitt, 2010; Parfitt, 1994) of osteoclasts and their precursors make it challenging to study them. Furthermore, there is no consensus on parameters such as seeding density (Kozbial et al., 2019) and supplement concentration (Mather, 1998; Shahdadfar et al., 2005) which greatly affect *in vitro* results. In this study, we investigated the effect of osteoclastic supplement concentration and priming in monocyte cultures and seeding density in PBMC and monocyte cultures on osteoclastogenesis, TRAP release over time and osteoclastic resorption and showed that these outcome measures not always tell the same story and may even contradict each other.

### 4.1 Osteoclastic supplement concentration correlated with osteoclastogenesis and resorption

Concentrations of 25 ng/mL of both osteoclastic supplements were sufficient to achieve the classical osteoclast-like appearance (Buckley et al., 2005). Higher concentrations did not lead to a proportional increase of large multinucleated cells. This is not unexpected, because the typical osteoclast appearance was already reached at this concentration. Resorption though did show a continuing increasing trend correlating with osteoclastic supplement concentration, confirming that the supplements are not only necessary for differentiation, but also for osteoclastic resorption. Surprisingly, TRAP hardly increased with higher concentrations of osteoclastic supplements, although lack of these supplements in the control group ensured a sharp decrease in TRAP release as expected. This matches the findings of Halleen, who proposed that TRAP release is indicative of osteoclast number but not activity (Halleen et al., 2006). This would suggest that while increasing osteoclastic supplement concentration above the threshold of 25 ng/mL not necessarily increases the number of osteoclasts, their resorptive capacity still increases. Priming is known to trigger gene expression (Hayes and Zoon, 1993) and benefit osteoclastogenesis (De Vries et al., 2015; Ross, 2006; Xu and Teitelbaum, 2013). Both TRAP release and resorption were lower without priming compared with the same supplement concentrations with priming. This confirmed that priming is a vital step in obtaining functionally competent osteoclasts, and suggests that priming, in contrast to increasing the osteoclastic supplement concentration above a necessary threshold, does lead to more osteoclasts.

### 4.2 Monocyte seeding density affects osteoclastogenesis, but not TRAP release or resorption after a certain threshold

The number of large Iβ3 or TRAP-positive cells increased with monocyte seeding density, but remarkably, the number of these cells with ≥3 nuclei hardly changed. TRAP release and resorption did not significantly increase with higher seeding density either, contrasting the findings of Halleen (Halleen et al., 2006). Resorption between all seeding densities showed no significant differences and TRAP analysis even suggested an inverse relationship between seeding density and TRAP release. These results suggest that there is an optimal seeding density (or range) for these cells which likely was superseded in the lower seeding densities already. What exactly was the limiting factor preventing the increase of TRAP release and resorption is not clear, but there are several possibilities. A seeding density too high could lead to competition for available surface area or specific medium components. Sharing an implicit shortage of available osteoclastic supplements or other media components with more competing cells could result in a lower availability per cell, with a higher proportion of cells not reaching the required threshold to differentiate or resorb. This would mean that commonly accepted culture conditions (Gstraunthaler et al., 1999; Place et al., 2017) might not be suitable for osteoclast cultures at these seeding densities. Lastly, the presence of non-resorbing osteoclasts (Karsdal et al., 2007), or a non-proportional relationship between osteoclast size and resorptive capacity could also have contributed to these findings. While large osteoclasts generally show more resorptive capacity than small ones (Lees et al., 2001), it could be that a large number of small osteoclast-like cells resorbed similar quantities as a lower number of large ones. This was supported by the presence of many slightly enlarged TRAP positive cells in the lower seeding densities, but only relatively few large osteoclasts in the higher seeding densities. These findings suggest that increased seeding densities do not necessarily result in larger or more osteoclasts, and that there is a plateau in seeding density above which no increase in osteoclastic resorption is seen anymore, that depending on the donor, could fall within commonly used seeding densities.

### 4.3 PBMC seeding density affects osteoclastogenesis and resorption

PBMCs are commonly used as a monocyte source. However, PBMCs have shown to be able to support osteoclastogenesis independent of external supplements (De Vries et al., 2019; Salamanna et al., 2016). Excluding these cells may result in osteoclasts that are biochemically different from their *in vivo* counterparts, despite showing the correct markers and resorption *in vitro*. In this study, higher seeding densities of PBMCs led to more and larger TRAP- or Iβ3-positive cells although very few cells had ≥3 nuclei. A gradual increase in resorption was seen, but TRAP release did not show a significantly increasing pattern over time, despite various significant differences at individual time points similar to those reported by others (Stessuk et al., 2021). Because the number of large TRAP-positive cells and resorption did seem to follow an increasing trend, it is unlikely that there was a competition for surface area or medium components. While seeding densities were double, the absolute number of monocytes per well was expected to be between 20 and 40 % of those seeded in the monocyte experiment (Kleiveland, 2015), suggesting that the number of monocytes per surface area could still be limiting in that experiment. Similarly, non-specific medium components likely were not the limiting factor in the monocyte experiment, but osteoclastic supplements could have been, considering the absolute difference in monocyte numbers between the monocyte and PBMC experiments. Resorption in the PBMC experiment increased with seeding density but reached only 10 % of that in the monocyte experiment despite having 20–40 % of the monocytes. Similarly, large TRAP-positive cells were seen, but the number of osteoclasts with ≥3 nuclei was proportionally low. This discrepancy could be explained by between-donor variation (Flanagan and Massey, 2003; Susa et al., 2004) or by the spatial constraints inherent to their cell fusion-based differentiation. At lower densities, the relationship between seeding density and both differentiation and resorption is likely closer to exponential than to linear, while the monocyte experiment suggested a maximum effective seeding density. Therefore, we propose that this relationship follows a logistic or S-shaped curve over the full spectrum of seeding densities. These findings suggest that PBMCs can be seeded at much higher densities than monocytes and still see increases in osteoclast formation and resorption, although the total amount of resorption is less-than-proportionate to the number of expected monocytes in the PBMC population.

### 4.4 Method recommendations

This study has shown that fluorescence imaging with either TRAP or Iβ3 effectively labels osteoclasts, although TRAP is expressed in undifferentiated mononuclear cells and monocytes as well (Park et al., 2012; Toyosaki-Maeda et al., 2001). Iβ3 staining appeared to exclusively target differentiated or differentiating multinucleated cells in these culture conditions and can thus be just as valuable for identifying osteoclasts (Husch et al., 2021). However, Iβ3 also stained many cells with only two visible nuclei, which means that Iβ3 staining alone is still not sufficient for identifying osteoclasts. The visual estimation of number and size of osteoclasts did not accurately correlate to resorption, and therefore should not be used to estimate osteoclastic activity. TRAP release was shown to increase with expected osteoclast cell numbers over time (Rucci et al., 2019), but could not be used as a quantitative marker of resorptive activity, although both supporting (Stessuk et al., 2021) and contradicting reports are available of this observation (Husch et al., 2021). While this was unexpected and diminishes the value of TRAP as an osteoclastic activity marker, TRAP still has a vital role as it can be accurately monitored in the cell culture supernatant.

### 4.5 Limitations and future research

The seeding densities of monocytes appeared too high to accurately show the effects of increases in seeding density despite being based on literature. Counting the number of alive and dead cells in culture supernatant or even further analyzing them with flow cytometry could shed light on what caused the observed plateau in resorption. The ratio of the monocyte subtypes was not investigated in this study but would be required when comparing different donors with each other. Although given the experimental conditions used it is highly probable that the cells labeled as osteoclast are in fact osteoclasts, all common markers used for osteoclasts could also indicate the presence of multinucleated giant cells (MNGCs) instead. PCR or ELISA analysis of Cathepsin K, calcitonin receptor, RANK (OC markers), CD86 or HLA-DR (MNGC markers) would provide additional certainty thereof (Miron et al., 2016). Quantitative resorption analysis on osteo assay plates was done using image analysis software, but due to lighting, stitching and contrast artifacts we were not able to accurately analyze complete wells. Instead, a same-size-for-all rectangle in the center of the well was analyzed. This resulted in missed resorption spots along the edges of the wells. Ideally, a method should be developed that can reliably analyze the complete well, possibly through additionally using a staining technique such as von Kossa. A limitation of these osteo assay plates is that they contain only a mineralized coating and lack a collagenous extracellular matrix, limiting the assay to mineral dissolution and neglecting enzymatic matrix cleaving. The golden standard of using bone or dentine discs do provide this, but instead are much less reliable for resorption quantification. Both osteoclastic supplements were used in equal concentrations in the osteoclastic supplement concentration experiment, with the risk of overestimating the concentration needed of one supplement, or underestimating the concentration needed of the other. Considering the limited number of cells available per donor, it was not feasible to test all unequal combinations of supplements as well. Osteoclast cultures show a large between-donor variation (Susa et al., 2004). This means that any concrete number derived from these experiments is valid only for cells from this donor.

## 5 Conclusion

In this study, we have shown that common non-standardized culturing conditions of monocyte or peripheral blood mononuclear cells had a significant effect on the *in vitro* generation of functional osteoclasts. We showed how increased osteoclastic supplement concentrations support osteoclastic differentiation and resorption but not TRAP release, while priming resulted in increased TRAP release as well. Increased monocyte seeding densities resulted in more and larger TRAP positive cells, but not in more resorption or TRAP release. Increasing PBMC seeding densities resulted in more and larger TRAP positive cells and more resorption, although both the number of cells with ≥3 nuclei and resorption were disproportionally low compared to the monocyte seeding density experiment. Exploration of commonly used markers for osteoclast cultures demonstrated that Iβ3 staining was an excellent osteoclast marker in addition to TRAP staining, while supernatant TRAP measurements could not accurately predict osteoclastic resorptive activity. With improved understanding of the effect of seeding density and osteoclastic supplement concentration on osteoclasts, experiments yielding higher numbers of functional osteoclasts can ultimately improve our knowledge of osteoclasts, osteoclastogenesis, bone remodeling and bone diseases.

The following is the supplementary data related to this article.

## CRediT authorship contribution statement

**Stefan J. A. Remmers:** Conceptualization, Data curation, Formal analysis, investigation, methodology, software, project administration, resources, supervision, validation, visualization, writing – Original Draft Preparation, writing – Review & Editing. **Freek C. van der Heijden:** Data curation, Formal analysis, investigation, methodology, software, validation, visualization, Writing – Review & Editing. **Keita Ito:** Conceptualization, project administration, resources, supervision, validation, Writing – Review & Editing. **Sandra Hofmann:** Conceptualization, funding acquisition, project administration, resources, supervision, validation, Writing – Review & Editing.

## Declaration of competing interest

All authors declare that they have no conflicts of interest.

## Data Availability

Data will be made available on request.
